# Frequency of concomitant injuries in maxillofacial trauma in a tertiary health care centre in India: A 5-year retrospective study

**DOI:** 10.1016/j.cjtee.2024.03.008

**Published:** 2024-03-22

**Authors:** Saubhik Dasukil, Shiwangi Verma, Ashok Kumar Jena, Mounabati Mohapatra

**Affiliations:** Department of Dentistry, All India Institute of Medical Sciences, Sijua, Bhubaneswar, Odisha, India

**Keywords:** Maxillofacial trauma, Associated hard tissue injuries, Associated soft tissue injuries, Road traffic accident

## Abstract

**Purpose:**

Road traffic accidents (RTA), assaults, falls, and sports-related injuries are the leading causes of maxillofacial trauma. Due to quite different geographical environment and fast urbanization, the use of various protective equipment is restricted in India. Thus, compared to other countries, there might be a significant difference in the pattern and frequency of associated injuries among subjects with maxillofacial trauma. The present study was conducted to identify the causes and pattern of various maxillofacial fractures and the frequency of other related injuries among subjects with maxillofacial trauma.

**Methods:**

This is a cross-sectional retrospective study recording 2617 subjects with maxillofacial trauma from October 2017 to October 2022. The patient demographics, causes of trauma, types of maxillofacial injury, and associated soft and hard tissue injuries were recorded. The types of maxillofacial and associated injuries were diagnosed from details of clinical examinations and the interpretation of various radiographs available in the file. The associated injuries were divided into head injury, other bony injuries, and soft tissue and vital structure injuries. Descriptive statistics and the test of proportion were used. A *p* value < 0.05 was considered as a level of significance.

**Results:**

The maxillofacial injuries were significantly common in patients aged 16 – 45 years (66.7%) than in patients aged ≤ 15 and > 46 years (33.3%) (*p* < 0.001). The RTA was the most common cause of maxillofacial injury (*n* = 2139, 81.7%), followed by fall (*n* = 206, 7.9%), other causes of injury (*n* = 178, 6.8%), and assaults (*n* = 94, 3.6%). The maxillofacial injury by 2-wheel vehicle accidents was significantly higher than that by 4-wheel vehicle and other vehicle accidents (*p* < 0.001). There was a significant correlation between alcohol and RTA (*p* < 0.001). The head injury (*n* = 931, 61.1%) was the most common associated injury, followed by soft tissue and vital structures injuries (*n* = 328, 21.5%) and other bone injuries (*n* = 264, 17.3%).

**Discussion:**

Head injury was the most common associated injury followed by soft tissue and vital structures and bone injuries among subjects with maxillofacial trauma. Clavicle fracture and injury to the lower extremities were the most common hard and soft tissue-associated injuries.

## 1 Introduction

Associated soft and hard tissue injuries among subjects with maxillofacial trauma are common^1^, and their early identification is crucial for patient management. The road traffic accidents (RTAs), assaults, falls, and sports-related injuries are the leading causes of maxillofacial trauma. In developed countries, assault is the most predominant cause, while RTA remains the most frequent cause in developing countries.^2^ The World Health Organization in 2018 identified speed, alcohol, seat belts, helmets, child restraint seat, and visibility as 5 major risk factors of the RTA.^3^ Drunk driving is also one of the significant causes of the RTA, which is widespread among commercial vehicle drivers in India.^4^ Also, the correct use of helmet can lead to 42% of reduction in the risk of fatal injuries and 69% of reduction in the risk of head injuries.^3^ Literature reveals that the most common associated injuries among subjects with maxillofacial trauma are head injury. The commonly affected fracture sites are nasal bone and zygomatic orbital complex, followed by the mandible.^5^ However, in India, a vast difference in its topography and increased urbanization lead to the restricted use of various protective equipment. Thus, there might be a significant difference in the pattern and frequency of associated injuries among subjects with maxillofacial trauma compared to other countries. Although many studies described the nature of various maxillofacial injuries, to the best of our knowledge, there are very few studies reported with such a large number of subjects for evaluating the nature of associated injuries. Thus, the present study was conducted to identify the causes and pattern of various maxillofacial fractures and other related injuries among subjects with maxillofacial trauma. Updated knowledge of the etiology, fracture pattern, and the relationship between the severity of the maxillofacial injury and concomitant injuries could help in early diagnosis and effective treatment of patients in which further focus required.

## 2 Methods

This cross-sectional retrospective study was carried out in the department of dentistry and department of trauma and emergency, in a tertiary care hospital in the Eastern part of India. The file records of all the subjects with maxillofacial trauma between October 2017 to October 2022 were examined. A standard proforma was used to record various data, including the patient's demography, causes of trauma, types of maxillofacial fractures, and associated soft and hard tissue injuries.

### 2.1 Grouping

The cause of maxillofacial trauma divided into 4 groups, i.e. RTA, assaults, fall, and others. Being run over by motorcycles, bicycles, and automobiles were included in RTA; injuries by domestic violence and physical fighting were considered as assaults; fall on the ground or fall from height were included in fall group; and others group included accident with animals, sports-related injuries, hit by an object, industrial injuries and gunshot injuries, etc. The diagnosis of maxillofacial trauma type was confirmed based on clinical examinations in the patient's record file and various radiographs.

The associated injuries were divided into 3 groups i.e., head injury, other bone injuries, and soft tissue and vital structure injury. Subjects with no associated injuries considered as isolated maxillofacial injury. Associated bone injuries included a fracture of clavicle, rib, femur, tibia, fibula, humerus, radius, ulna, cervical spine, pelvis, and others. Soft tissue injuries included scalp, ocular, neck, thorax, abdomen, upper and lower extremities, and nerve and vessel injuries.

### 2.2 Statistical analysis

A master file was created, and the data were statistically analyzed on a computer with SPSS (Statistical Packages for the Social Sciences, Chicago, IL; version 22) software. Descriptive statistics and the test of proportion were used for data analysis. Probability (*p* value) < 0.05 was considered as a level of significance.

## 3 Results

There were 2617 subjects with sustained maxillofacial trauma during the 5-year study period. The patients' age ranged from 12 to 73 years, and the median age was 26.4 years. The maxillofacial injury was significantly more common among the age of 16 – 45 years compared to the age of ≤ 15 and > 46 years (*p* < 0.001) (Table 1). The distribution of various causes of maxillofacial trauma was described in Table 1. The RTA was the most common cause of maxillofacial trauma, followed by fall, other causes of injury and assaults.

**Table 1:** Distribution of subjects and causes of maxillofacial trauma, *n* = 2617.

Parameters	*n* (%)	*p* value
Sex		< 0.001
Male	2084 (79.7)	
Female	533 (20.3)	
Age (year)		< 0.001
≤ 15	94 (3.6)	
16 – 30	1077 (41.2)	
31 – 45	669 (25.6)	
46 – 60	485 (18.5)	
> 60	292 (11.2)	
Causes		< 0.001
RTA	2139 (81.7)	
Fall	206 (7.9)	
Assault	94 (3.6)	
Others	178 (6.8)	

RTA: road traffic accident.

Analysis of various factors related to RTA revealed that maxillofacial trauma by 2-wheel vehicle accidents was significantly more common compared to 4-wheeler vehicle and other vehicle accidents (*p* < 0.001) (Table 2). Injury among subjects without a helmet while driving 2-wheel vehicle was significantly more than with helmet (*p* < 0.001). The use of seatbelt had no significant effect on the maxillofacial injury (*p* = 0.260) among RTA subjects associated with the 4-wheel vehicle accident (Table 2).

**Table 2:** Various factors associated with maxillofacial trauma among subjects with RTA.

Factors associated with RTA	*n* (%)	*Z* value	*p* value
Type of vehicle (*n* = 2139)			
Two-wheel vehicle	1865 (87.2)	11.03	< 0.001
Four-wheel vehicle	192 (9.0)		
Other vehicles	82 (3.8)		
Use of helmet in 2-wheel vehicle (*n* = 1865)			
Without helmet	1581 (84.8)	9.89	< 0.001
With helmet	284 (15.2)		
Use of seat belt in 4-wheel vehicle (*n* = 192)			
Without seatbelt	103 (53.6)	1.13	0.260
With seatbelt	89 (46.4)		

RTA: road traffic accident.

Among 2617 subjects with maxillofacial trauma, 1278 (48.8%) subjects had a history of alcohol use before the injury. Of the 1278 subjects, 1123 (87.9%), 114 (8.9%), 39 (3.1%), and 2 (0.2%) had RTA, assaults, fall, and others cause of maxillofacial trauma respectively. The correlation coefficient showed a statistically highly significant association between alcohol and RTA (*p* < 0.001).

The distribution of the pattern of maxillofacial fractures among studies subjects were described in Table 3. Isolated mandibular fracture, isolated zygomatic maxillary complex fracture, and pan facial fracture were the most common patterns. Of all the subjects with maxillofacial trauma (*n* = 2617), the associated injuries were present in 1523 subjects. Of 1523 subjects, head injury (*n* = 931, 61.1%, *p* < 0.001) was the most common among associated injury, followed by other soft tissue and vital structures injury (*n* = 328, 21.5%) and other bone injuries (*n* = 264, 17.3%). The detail analysis of other soft tissue and vital structure injuries were showed in Table 4, and the detailed associated bone injuries were showed in Table 5.

**Table 3:** The pattern of maxillofacial fractures among all studied subjects, *n* = 2617.

Types of maxillofacial fracture	*n* (%)
Isolated mandibular fracture	651 (24.9)
Isolated zygomatic maxillary complex fracture	576 (22.0)
Pan facial fracture	468 (17.9)
Isolated midface (Le Fort 1, 2, 3) fracture	365 (13.9)
Isolated nasal/NOE complex fracture	225 (8.6)
Isolated dento-alveolar fracture	129 (4.9)
Isolated frontal bone fracture	116 (4.4)
Isolated orbital fracture	87 (3.4)
Total	2617 (100.0)

NOE: Naso-orbital-ethmoid.

**Table 4:** The details of other soft tissue and vital structure injury among subjects with maxillofacial trauma, *n* = 328.

Other soft tissue and vital structures	*n* (%)
Lower extremity injury	97 (29.6)
Upper extremity injury	59 (18)
Neck injury	54 (16.5)
Ocular injury	37 (11.3)
Nerve and vessels injuries	35 (10.7)
Scalp injury	24 (7.3)
Thorax and abdomen injury	22 (6.7)
Total	328 (100.0)

**Table 5:** Frequency of associated bone injury among subjects with maxillofacial trauma, *n* = 264.

Associated bone injury	*n* (%)
Clavicle fracture	77 (29.2)
Femur fracture	43 (16.3)
Rib fracture	38 (14.4)
Tibia/fibula fracture	32 (12.1)
Humerus fracture	25 (9.5)
Radius/ulnar fracture	22 (8.3)
Cervical spine fracture	13 (4.9)
Pelvis fracture	3 (1.1)
Other fracture	11 (4.2)
Total	264 (100.0)

## 4 Discussion

The present study assessed the causes and various patterns of maxillofacial trauma and other associated injuries. We observed that the frequency of maxillofacial trauma was highest among the age of 16 – 30 years, because individuals between the age of 16 – 30 years are most active members of society. Similar to our observation, many authors also reported maximum maxillofacial trauma between 16 and 30 years of age in populations of United Arab Emirates (UAE) and Nigerian.^6^^,^^7^ In India, although the legal driving age is 18 years, there are no police patrolling and many adolescents below 16 years drive 2-wheel vehicle without driving license in many rural and semi-urban areas. This could be the reason that subjects had 3.6% maxillofacial trauma below the age of 16 years.

The present study revealed that males were 4 times more likely to experience maxillofacial trauma than females, due to the predominance of male automobile driver and the consumption of alcohol and other psychoactive substances. However, AI Ahmed et al.^6^ reported that in the UAE, the incidence of maxillofacial trauma was 11 times higher in males than in females, due to the fact that males had more freedom to work outdoors and engage in adventurous activities. For a better life, more family members work for extra income in developing countries. Therefore, in India, females are getting more works outdoor than in the UAE, which exposes them to more RTAs. In Jordan, females are more engaged in many social events, which results in more occurrence of maxillofacial trauma in females.^8^

We observed that RTA was the most common cause of maxillofacial trauma, similar to many previous studies conducted in India.^9^^,^^10^ The lack of adequate road safety measures by the government, road safety awareness among drivers, poor condition of roads, speed driving, drunk driving, not wearing helmet and seat belt, use of mobile phones while driving, and behavioral disorders are the reasons for RTA. We observed a significantly higher proportion of RTA associated with 2-wheel vehicle, particularly those without wearing a helmet. Similarly, we found that the maxillofacial injuries were more among individuals driving 4-wheel vehicle without wearing the seat belt. Therefore, compulsory preventive measures such as wearing a helmet and seat belt, enforcing the law regarding drunk driving, regulating people driving behaviors, and training people regularly of proper road safety guidelines are essential to reduce the frequency of fatal motor vehicles accidents. Similar to our results, RTA was also the most common cause of maxillofacial trauma in many other countries like Saudi Arabia, Jordan, and Iran.^6^, ^7^, ^8^ However, according to Arslan et al.,^11^ assault was the most common cause of maxillofacial trauma in Ankara (Turkey). Bakardjiev et al.^12^ also mentioned that assault was the most common cause of maxillofacial injury in the Bulgarian community. Alcohol abuse and increasing aggression in society was the probable reason for such difference.

The fall was the second leading cause of maxillofacial injury in the present study. Many previous studies also reported similar observations.^7^^,^^8^^,^^13^ Maxillofacial injuries due to falls showed bimodal age distribution i.e., under 10 years of the age and over 60 years of the age. Gerbino et al.^14^ reported that facial fractures were the most common secondary injury after falls in the elderly population. Interpersonal violence from increased use of alcohol and psychoactive drugs is becoming a significant problem in many countries like the USA.^1^ We found very less frequency of maxillofacial injury caused by interpersonal violence, because it will be managed at the primary healthcare center even if occasionally disclosed.

In accordance with the previous study, the mandible having the characteristics of prominence, mobility, multiple anatomical weak sites, and less structural support than the maxilla was thus the most common fracture site for maxillofacial trauma.^15^^,^^16^ However, Arslan et al.^11^ reported that the nasal bone was most frequently involved, whereas Dibaie et al.^17^ reported that zygomatic complex was the most predominant involved site. In contrast, we found isolated zygomatic complex fractures accounted for 22% and nasal bone fractures accounted for 8.6% of total cases.

Literature reveals that the associated injuries among subjects with maxillofacial trauma range from 8.3% to 99.3%.^1^^,^^18^, ^19^, ^20^ Being similar with other studies, 58.1% of subjects had associated injuries like head injury along with damage to other bone and soft tissue and vital structures in our study.^1^ Thus, proper clinical examination and multidisciplinary approach for trauma patients are essential for optimum diagnosis and management. Of 1523 subjects with associated injuries, 931 subjects had head injuries, which comprised skull fracture, traumatic hematoma, cerebral contusion, and concussion accounted. There is a wide variation in the frequency of head injury among subjects with maxillofacial trauma.^2^^,^^19^ Many previous studies also reported that head injury was the most common associated injury among subjects with maxillofacial trauma.^2^ Lim et al.^20^ reported that the frequency of head injury in subjects with facial bone fractures was only 5.4%, but Martin et al.^21^ and Hayter et al.^22^ reported 79.4% and 86% involvement of head injury, respectively. It is proposed that facial bones absorb the majority of traumatic forces and protect the brain from damage. The high frequency of head injury among subjects with maxillofacial trauma could be because of differences in the nature and cause of trauma.

Isolated soft tissue injury occurred in 21.5% of total subjects. The soft tissue injury in the lower extremities was the most common sites, followed by upper extremities, neck, and ocular area, in patients reported with maxillofacial trauma. Jha et al.^10^ also reported similar observations among maxillofacial trauma subjects in southern of India. There were associated bone injury among 17.3% of subjects. The clavicle because of its critical location was the most commonly involved bone in the maxillofacial trauma, especially due to a fall on the outstretched arm or direct force on the shoulder. Obuekwe et al.^23^ also reported that clavicle fracture was the most common associated bone fracture among patients with orthopedic injuries.

Optimum care of maxillofacial trauma patients demands precise detection of concomitant injuries. In the emergency room, however, the examination of polytrauma patients could be challenging, and the risk of under-diagnosis is high. Most of the time, triage and implementation priorities are determined by the severity of concurrent injuries based on relevant clinical findings.^24^ Thus a proper clinical examination and a multispecialty team approach to the trauma patient is essential for prime management.

Maxillofacial trauma is most common in people between the ages of 16 and 45 years, with male having 4 times more likely to have maxillofacial trauma than female. RTA was the leading cause of maxillofacial trauma, which had a very high correlation of alcohol. Maxillofacial trauma caused by 2-wheel vehicle was more common than by 4-wheel vehicle. Driving without wearing a helmet and seat belt was often associated with RTA. Head injury and injury to the other vital structures were the most common associated injuries among individuals with maxillofacial trauma. Clavicle fracture was the most associated bone injury among maxillofacial trauma subjects. The inherent potential of missing information in the patient's record file was the main demerit of the present study. To overcome the limitation, comprehensive document policies and invest in advanced electronic health record systems in future can be implemented.

## CRediT authorship contribution statement

**Saubhik Dasukil:** Conceptualization, Methodology, Investigation, Writing – Original Draft. **Ashok Kumar Jena:** Conceptualization, Methodology, Formal Analysis, Writing – Original Draft. **Mounabati Mohapatra:** Conceptualization, Methodology. **Shiwangi Verma:** Formal Analysis, Writing – Original Draft.

## Ethical statement

No ethical clearance required.

## Funding

Nil.

## Declaration of competing interest

All authors have declared no conflict of interest.
